# An End-to-End Depression Recognition Method Based on EEGNet

**DOI:** 10.3389/fpsyt.2022.864393

**Published:** 2022-03-11

**Authors:** Bo Liu, Hongli Chang, Kang Peng, Xuenan Wang

**Affiliations:** ^1^Department of Emergency, The Second Hospital of Shandong University, Jinan, China; ^2^School of Information Science and Engineering, Southeast University, Nanjing, China; ^3^Department of Rehabilitation Medicine, Guangzhou First People's Hospital, School of Medicine, South China University of Technology, Guangzhou, China; ^4^Shanghai Key Laboratory of Psychotic Disorders, Shanghai Mental Health Center, Shanghai Jiaotong University School of Medicine, Shanghai, China

**Keywords:** depression recognition, electroencephalogram (EEG), convolutional neural network (CNN), end-to-end, EEGNet

## Abstract

Major depressive disorder (MDD) is a common and highly debilitating condition that threatens the health of millions of people. However, current diagnosis of depression relies on questionnaires that are highly correlated with physician experience and hence not completely objective. Electroencephalography (EEG) signals combined with deep learning techniques may be an objective approach to effective diagnosis of MDD. This study proposes an end-to-end deep learning framework for MDD diagnosis based on EEG signals. We used EEG signals from 29 healthy subjects and 24 patients with severe depression to calculate Accuracy, Precision, Recall, F1-Score, and Kappa coefficient, which were 90.98%, 91.27%, 90.59%, and 81.68%, respectively. In addition, we found that these values were highest when happy-neutral face pairs were used as stimuli for detecting depression. Compared with exiting methods for EEG-based MDD classification, ours can maintain stable model performance without re-calibration. The present results suggest that the method is highly accurate for diagnosis of MDD and can be used to develop an automatic plug-and-play EEG-based system for diagnosing depression.

## 1. Introduction

Depression is one of the most prevalent mental disorders. Patients with depression experience a severely impaired quality of life and are at increased risk of suicide (1–3). Patients infected with COVID-19 experience sleep disorders and are at increased risk of anxiety or depression, all of which are psychological complications (4–7). Yet depression is frequently undiagnosed and untreated because of a lack of effective therapies and inadequate mental-health resources (8). The onset of depression is usually gradual, but can be abrupt, and its progression throughout life varies considerably. Symptoms of depression often occur along with emotional, neurovegetative, and cognitive symptoms, and since they are commonly present in other psychiatric disorders and medical conditions, detection of depressive syndrome is problematic.

Identification of effective biomarkers for major depression is of great importance for improving the diagnosis and effective treatment of this common and debilitating neuropsychiatric disorder. Several different treatments are currently available, including a wide variety of antidepressant drugs (9–11), electroconvulsive therapy (ECT) (12), repetitive transcranial magnetic stimulation (rTMS) (13), and deep brain stimulation (DBS) (14). However, half of patients with depressive disorder do not respond to current treatments. Therefore, it is necessary to discover new brain activity mechanisms and specific biomarkers for patients who respond to treatment in order to predict the onset and course of the disease, increase the therapeutic response, and enable detection of those patients who are resistant to individual therapies.

In recent years, use of non-invasive sensor-based methods, such as electroencephalography (EEG), has been widely reported in the literature (15, 16). One of the most remarkable research efforts has been in the area of efficient neural network-based approaches to analysis of EEG signals for automatic assessment of mental disorders such as major depressive disorder (MDD) and bipolar disorder (BD). Indeed, EEG is a non-invasive, effective, and powerful tool for recording the brain's electrical activity and diagnosing various mental disorders such as MDD, BD, anxiety (17), schizophrenia (18), and sleep disorders (19). In the case of depression, the body releases signals into the brain that affect neuronal production and communication, which slows or otherwise changes some regions of the brain. Variations in voltage resulting from changes in ionic current within the brain's neurons contribute to EEG signals and might help to diagnose mental disorders like depression. Development of robust approaches to analysis of brain signals is challenging because of their complexity and significant variability related to age and mental state. Moreover, EEG signals are frequently affected by different types of noise due to eye blinking and body motion (20). It is needed a deep learning technique that can effectively learn brain activity patterns from EEG signals.

To achieve the above requirements, we present a novel end-to-end architecture, supervised EEG-based event-related potential (ERP) classification. The EEG database used here is small and does not require complex EEG pre-processing. This method not only successfully extracts information across different subjects for ERP decoding, but also accomplishes three tasks simultaneously.

The remainder of this article is structured as follows: We firstly provide background and introduce the database, then we describe the structure of the proposed method, finally, experimental results are presented and discussed.

## 2. Materials and Methods

**Figure 1:**
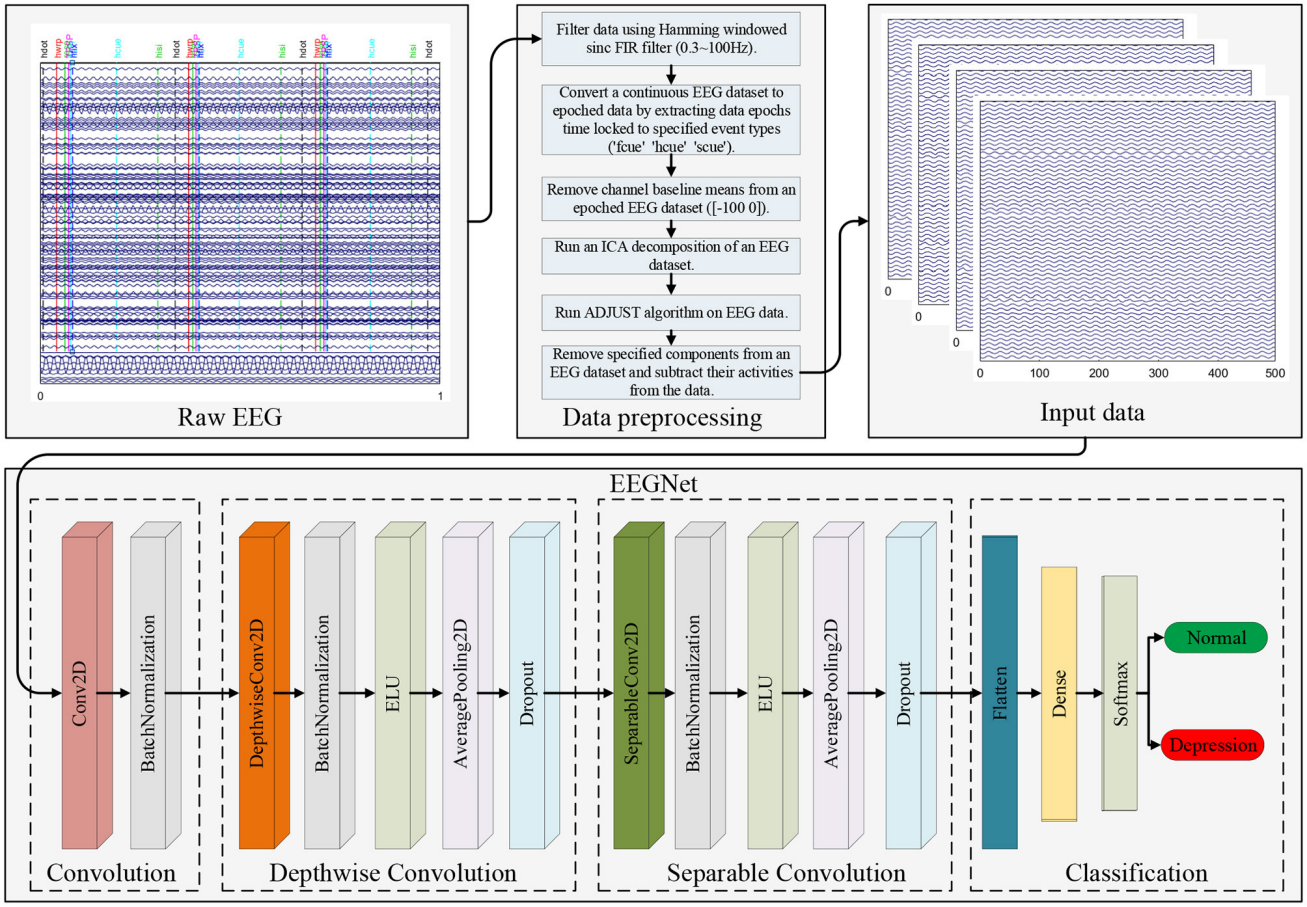
An EEG End-to-End Depression Recognition Framework.

This section introduces the EEG depression database, signal pre-processing, evaluation metrics, and details of EEGNet and how it can be used to recognize depression. The end-to-end depression recognition framework is shown in Figure 1.

### 2.1. Depression Database

**Figure 2:**
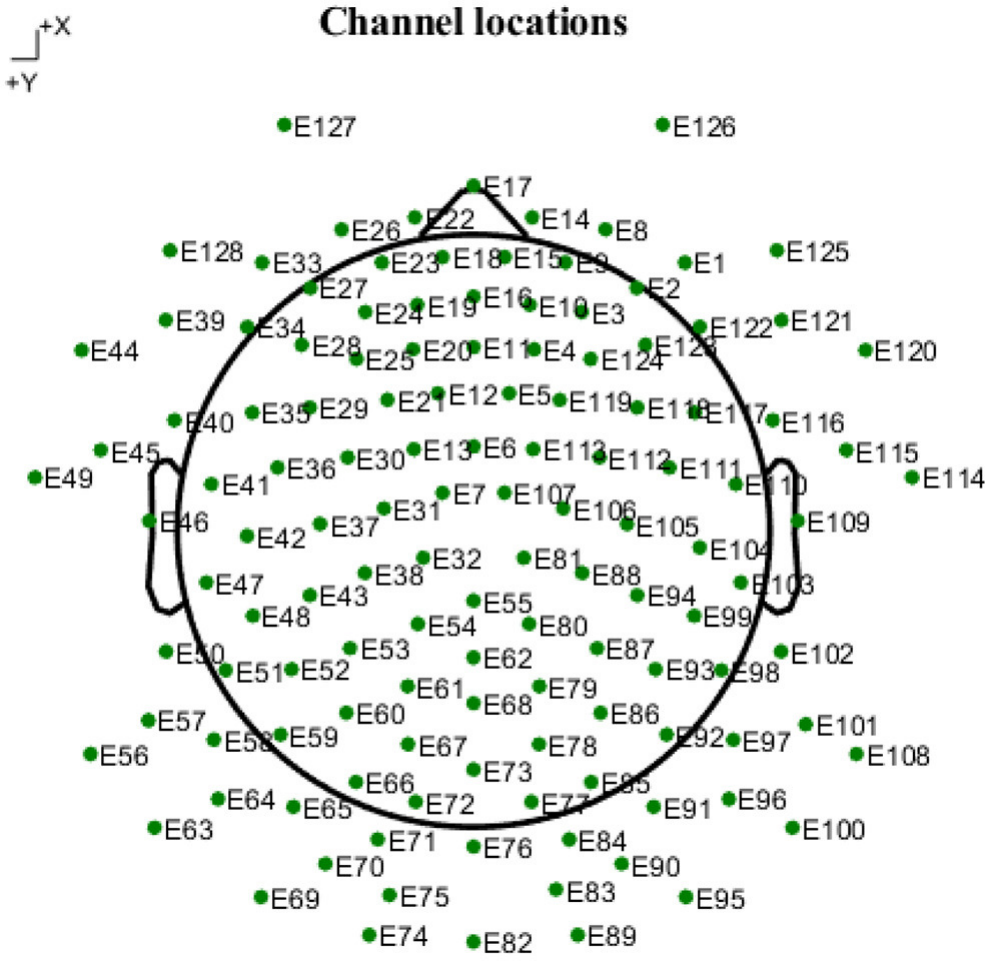
Topological structure map of 128-electrode channels mapped to a two-dimensional picture. The circle represents the electrode, and the label inside is the serial number and name of the electrode.

For depression recognition, we chose the multi-modal open dataset for mental-disorder analysis, i.e., the MODMA dataset. The dataset included 128-channel ERP recordings in Figure 2, from 24 subjects with MDD and 29 healthy controls (HCs) in the age range of 16–52 years (21–23). The sampling frequency was 250 Hz. The ERP experiment was a dot-probe task, and its cue stimuli included three kinds of emotional-neutral face pairs, namely happy-neutral (Hcue), sad-neutral (Scue), and fear-neutral (Fcue). In the formal experiment, subjects sat 60 cm in front of a monitor and were asked to focus on the emotion-neutral face pairs randomly presented as targets at the left and right positions. When these face dots appeared, the subjects were asked to press buttons on the reaction box as quickly and accurately as possible; they rested after completing each module. The task consisted of three parts (Hcue, Fcue, and Scue), each with 160 trials. At the beginning of each trial, a fixed white cross appeared on the center of the screen, starting at 300 ms and continuing throughout the experiment. Emotional-neutral pairs of face stimuli were presented on the screen as 500 ms cues, and the faces were arranged in a pseudo-random order. After a short interval (about 100–300 ms), the point probes randomly appeared at the left and right positions of the fixed cross for 150 ms. At the same time, participants were asked to identify the location of the points and to record their responses by pressing a button on the reaction box with their index finger. If the system did not receive responses within 2 s, participants would be directed to a subsequent trial and a black screen was then displayed for 600 ms. This process proceeded gradually until a block was completed. Each block was repeated until the entire task was complete. The entire experimental task was finished in 25 min.

### 2.2. Pre-processing Engineering

We used EEGLAB toolbox in MATLAB to preprocess the raw data as follows Brunner et al. (24): (1) an EEG dataset was converted to an average for reference; (2) the data were filtered using a Hamming-windowed sinc FIR filter (0.3–100 Hz) to remove the 50 Hz power interference; (3) the continuous EEG dataset was converted to epoched data by extracting data epochs that were time-locked [-100 500]to specified event types (Hcue, Fcue and Scue); (4) channel baseline [-100 0] means were removed from the epoched EEG dataset; (5) an independent component analysis decomposition of the EEG dataset was run and specified components were removed; and (6) their activities were subtracted from the EEG dataset using the Adjust algorithm, as shown in Figure 1. The format of the preprocessed data is [*trials, channels, samples, kernels*], where *trials* = 480, *channels* = 128, *samples* = 125, *kernels* = 1.

### 2.3. EEGNet for Depression Recognition

EEGNet is a compact convolutional neural network (CNN) architecture that can be trained with minimal data to extract neurophysiologically interpretable features. A visualization and complete description of the EEGNet model are shown in Figure 1 and Table 1. It primarily included four blocks: convolution, depthwise convolution, separable convolution, and classification.

**Table 1:** EEGNet model structure and parameters.

**Layer (type)**	**Size**	**Output Shape**	**Param #**
input_1 (InputLayer)		(None, 128, 125, 1)	0
conv2d (Conv2D)	8#(1,100)	(None, 128, 125, 8)	800
batch_normalization (BatchNormalization)		(None, 128, 125, 8)	32
depthwise_conv2d (DepthwiseConv2D)	(128,1)	(None, 1, 125, 16)	2048
batch_normalization_1 (BatchNormalization)		(None, 1, 125, 16)	64
activation (Activation)	elu	(None, 1, 125, 16)	0
average_pooling2d (AveragePooling2D)	(1,4)	(None, 1, 31, 16)	0
dropout (Dropout)	0.5	(None, 1, 31, 16)	0
separable_conv2d (SeparableConv2D)	16#(1,16)	(None, 1, 31, 16)	512
batch_normalization_2 (BatchNormalization)		(None, 1, 31, 16)	64
activation_1 (Activation)	elu	(None, 1, 31, 16)	0
average_pooling2d_1 (AveragePooling2D)	(1,8)	(None, 1, 3, 16)	0
dropout_1 (Dropout)	0.5	(None, 1, 3, 16)	0
flatten (Flatten)		(None, 48)	0
dense (Dense)	2	(None, 2)	98
softmax (Activation)		(None, 2)	0
Total params: 3,618			
Trainable params: 3,538			
Non-trainable params: 80			

In the convolution block, we fitted eight 2D convolutional filters of size (1, 100), outputting eight feature maps containing the EEG signal at different band-pass frequencies. Then we added a layer for batch normalization to make the training process more stable and reduce overfitting (25).

As the convolutions in the depthwise convolution block were not fully connected to all previous feature maps, we used a depthwise convolution of size (128, 1) and depth = 2 to learn a spatial filter, which reduced the number of trainable parameters that required fitting. When this operation was used for EEG depression recognition, it provided a direct way to learn spatial filters for each temporal filter and enabled efficient extraction of frequency-specific spatial features. We applied batch normalization along the feature map dimension before applying exponential linear unit (ELU) nonlinearity. Then, we used a dropout layer of probability = 0.5 to help regularize and an average pooling layer of size (1, 4) to reduce the sampling rate.

In the separable convolution block, we used a separable convolution with a depthwise convolution of size (1, 16) followed by a pointwise convolution. The main benefit of separable convolutions is a reduction in the number of parameters that require fitting and explicitly decoupling the relationships within and between feature maps by first learning a kernel that summarizes each feature map, which optimally combines the outputs. When it was used for EEG-specific applications, this operation guided the summarizing of individual feature maps in time (depth convolution) and their optimal combination (pointwise convolution). This operation was also perfectly suited to EEG signals, since different feature maps can represent informative data over different time scales. In addition, average pooling layers of size (1, 8) were used for dimensionality reduction.

In the classification block, multi-dimensional features were downsampled to one dimension and directly passed to a softmax classification with 2 units to identify two categories, normal and depression.

### 2.4. Evaluation Index and Experimental Settings

**Figure 3:**
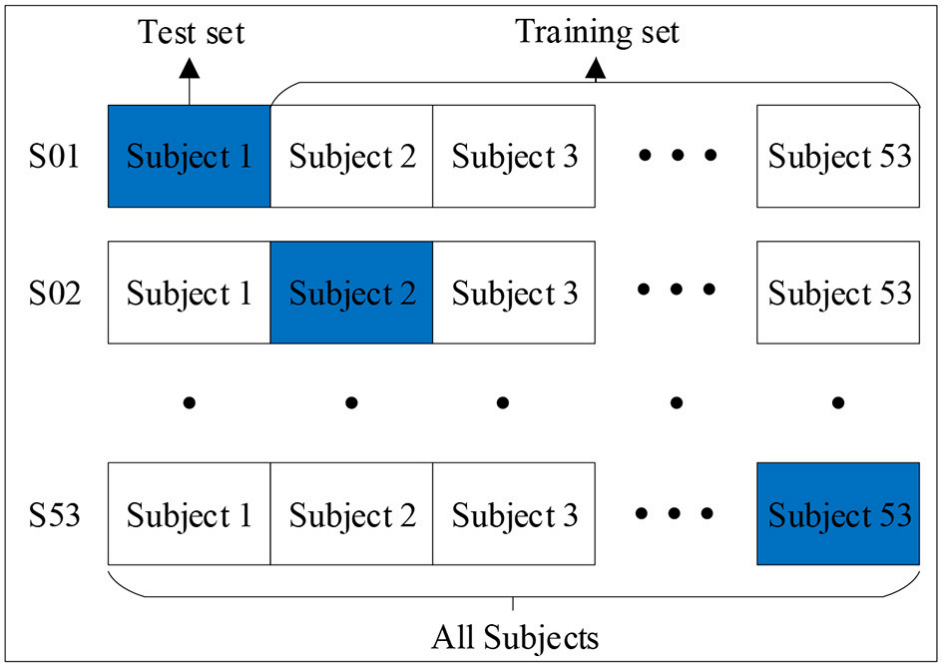
Leave-One-Subject-Out Cross-Validation.

We adopted the leave-one-subject-out cross-validation (LOSOCV) method to separate the training set from the validation set. Specifically, the training set was used to train the model, and the validation set was used to evaluate its generalization ability, as shown in Figure 3. The subject data was divided into 53 folds, with each representing the complete dataset of a subject. This protocol was suitable for small databases, could be trained by almost all the data, and was tested using one dataset. The experiment had no random factors, and the entire process was repeatable.

After modeling, several indicators were needed to measure the generalization ability of the model and further adjust the parameters to gradually optimize the model. As shown in Table 2, the first indicator was the confusion matrix. When the model diagnosed a normal person as normal, it was a true positive (TP); otherwise it was a false negative (FN). The model assigned a true negative (TN) when it diagnosed a depressive patient with depression; otherwise it was a false negative (FN). Five additional indicators were used to evaluate model classification, including (1) Accuracy: the proportion of correct to total samples, with each category treated equally; (2) Precision: the correct proportion of the positive samples predicted by the classifier; (3) Recall: the proportion of correctly predicted positive samples to all positive samples; (4) F1-Score: precision and recall affected each other, so in order to balance the two indicators and take into account the category imbalance, the weighted harmonic mean of precision and recall was used; (5) Kappa coefficient: an index used to test for consistency, usually to measure the effect of classification. Consistency was a measure of whether the model's predicted result was consistent with the actual classification result. The value range of all these indicators was [0, 1]. The larger the value, the better the predictive ability of the model.

**Table 2:** Confusion Matrix and Evaluation Index.

**Confusion Matrix**		**Predicted Label**	
		**Normal**	**Depression**
**True Label**	**Normal**	**True Positive (TP)**	**False Negative (FN)**
	**Depression**	**False Positive (FP)**	**True Negative (TN)**
Evaluation Index	(1) $\text{Accuracy}=\frac{TP+TN}{TP+TN+FP+FN}$.		
	(2) $\text{Precision}=\frac{TP}{TP+FP}$.		
	(3) $\text{Recall}=\frac{TP}{TP+FN}$.		
	(4) $\text{F1-Score}=\frac{2\times Precision\times Recall}{Precision+Recall}$.		
	(5) $\text{Kappa}=\frac{P_{a}-P_{e}}{1-P_{e}}$, $P_{a}=\frac{TP+TN}{TP+TN+FP+FN}$,		
	$P_{e}=\frac{\left(TP+FP\right)\left(TP+FN\right)+\left(FN+TN\right)\left(FP+TN\right)}{{\left(TP+TN+FP+FN\right)}^{2}}$.		

We fitted the Adam optimizer model, minimizing the categorical cross-entropy loss function. We ran 50 training iterations (epochs), performed validation stopping and saved the model weights, which produced the lowest validation set loss. During model training, the data was divided into training and validation sets using the train_test_split() function in the Python sklearn library, with the validation set assigned a proportion of 0.3.

## 3. Results and Discussion

Four types of experiment in which EEG signals were collected were classified by the type of face-pair stimulus used.

Experiment 1 (All): Subjects were stimulated by all three types of face pairs (480 trials for each subject);
Experiment 2 (Fcue): Subjects were only stimulated by fear-neutral face pairs (160 trials for each subject);
Experiment 3 (Scue): Subjects were only stimulated by sad-neutral face pairs (160 trials for each subject);
Experiment 4 (Hcue): Subjects were only stimulated by happy-neutral face pairs (160 trials for each subject).

### 3.1. Recognition Scores for End-to-End Recognition of Depression

**Figure 4:**
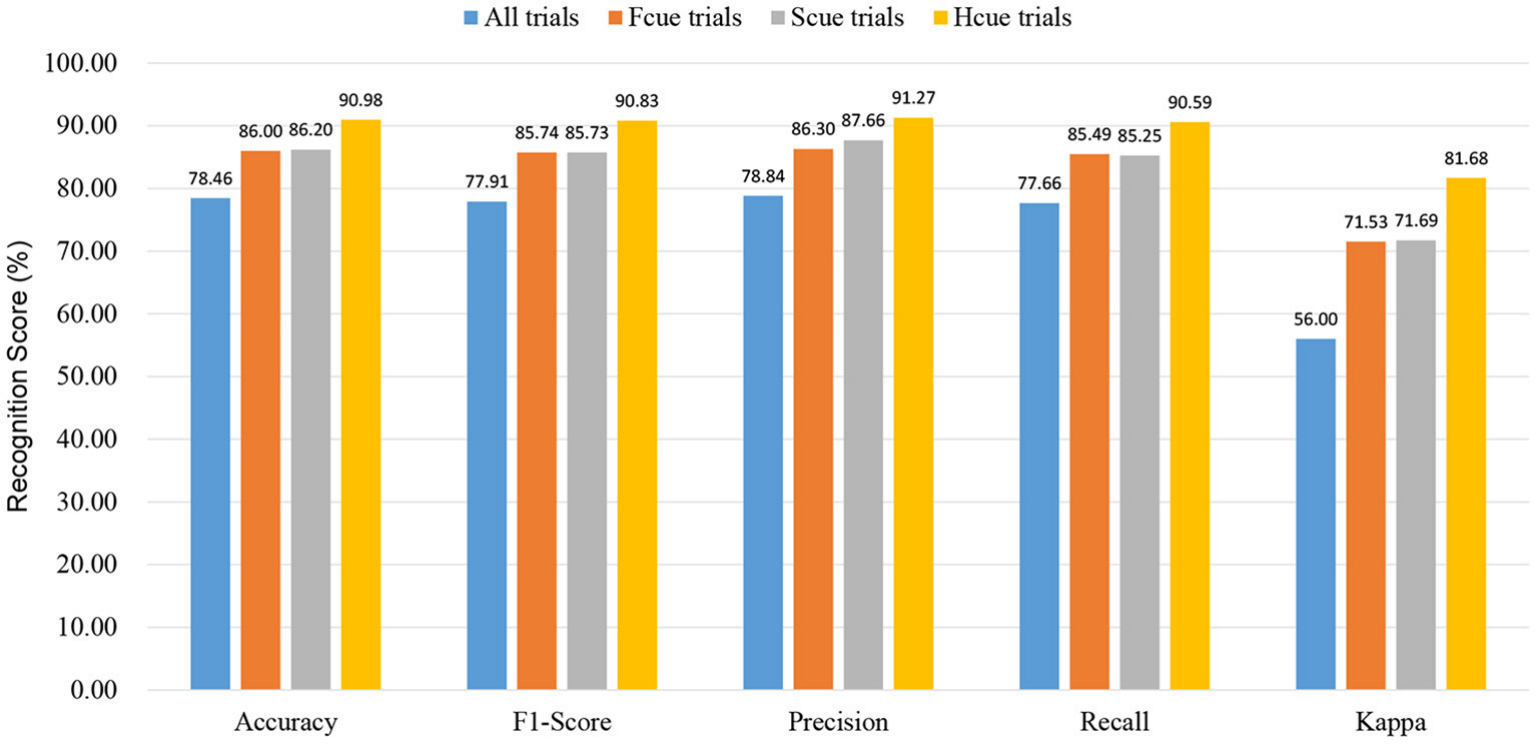
Recognition scores of end-to-end depression recognition.

Figure 4 lists the average values of the five metrics (accuracy, F1 score, recall, precision, and kappa) for the four sets of experiments. With the preprocessed signal used as input, the highest average classification accuracy (90.98%) obtained by LOSOCV was for Experiment 4. Similarly, the values of the other four indicators (F1 score: 90.83%; recall: 91.27%; precision: 90.59%; kappa: 81.68%) were all highest in Experiment 4 (i.e., Hcue trials). The scores from the experiments using the fear-neutral and sad-neutral face pairs were similar, with both being significantly lower than for the Hcue trials. Therefore, happy-neutral face pairs can be used as emotion-evoking materials to effectively discriminate between MDD patients and HCs. However, all the recognition scores in Experiment 1 were low, indicating that brainwaves may depend on the type of stimulus, misleading the network model and thus failing to distinguish depression from normal.

### 3.2. Accuracy of Experimental Results for Each Subject

**Figure 5:**
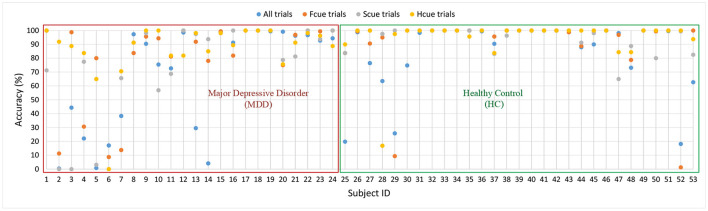
Scatter plot of four experimental results (Accuracy) for each subject.

In order to analyze the performance of the model, the accuracy of the four types of experiment is shown for each subject in Figure 5. The first 24 subjects in the figure experienced MDD, and subjects 25–53 were HCs. The scatter plot shows the distribution of the four experimental results. Among the MDD subjects, the correct rate for the first seven subjects was relatively low and, except for one experiment, those for subjects 8–24 were above 70%. The results show that recognition was better for HCs than for MDD subjects, and that Experiment 4 (Hcue) elicited results that were better than for the other experiments.

### 3.3. Confusion Matrix

**Figure 6:**
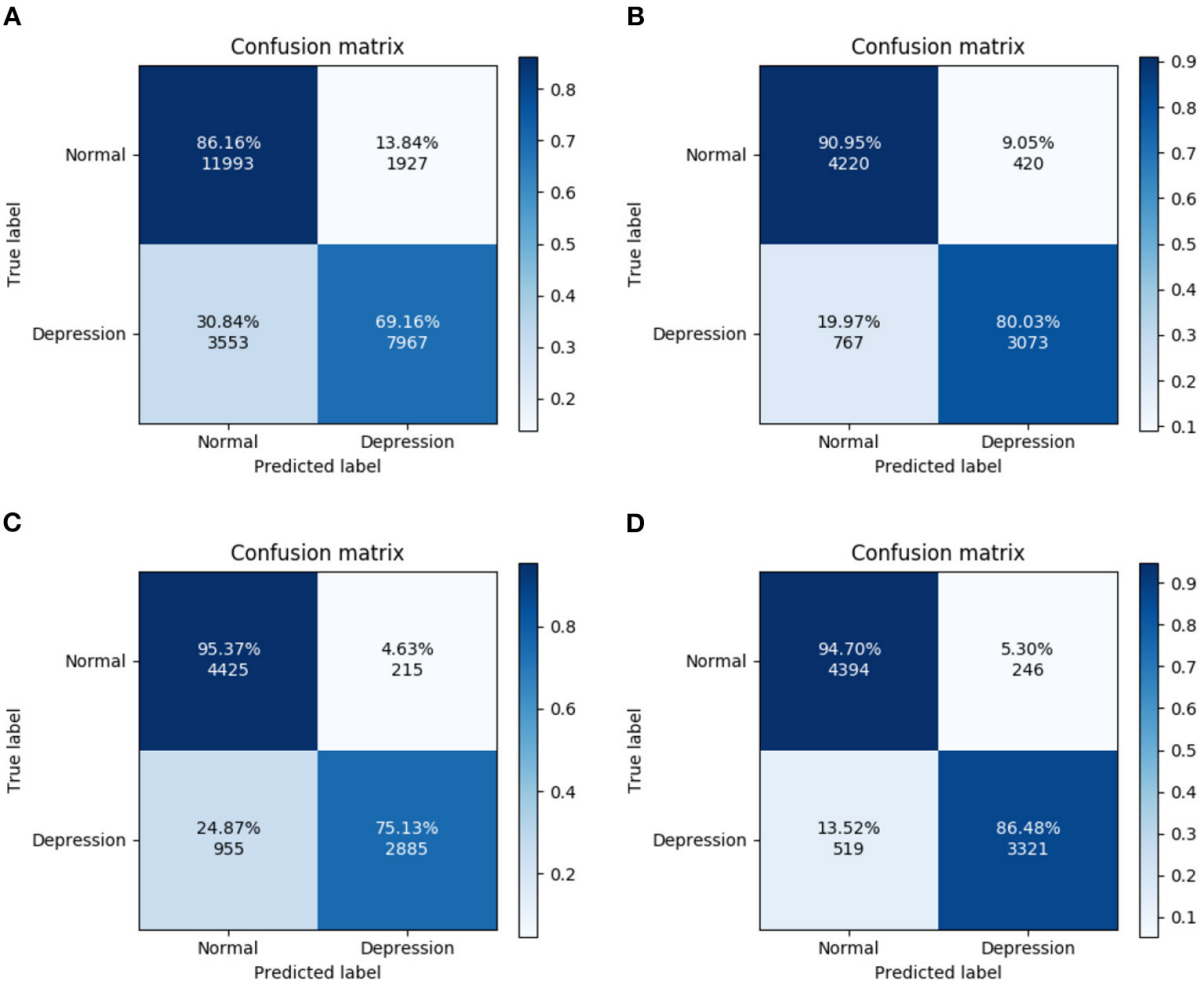
Confusion matrix for four experiments. **(A)** All trials, **(B)** Fcue trials, **(C)** Scue trials, and **(D)** Hcue trials.

Figure 6 shows that the highest recognition rate for the confusion matrix (95.37%) was that for HCs from Experiment 3. The highest recognition rate for MDD patients was 86.48% from Experiment 4. Differences in the number of MDD and HC subjects in the database (depressed: 24; normal: 29) and the small number of categories in this model may account for poor learning in some experiments and slightly different recognition rates.

### 3.4. Model Optimization

**Figure 7:**
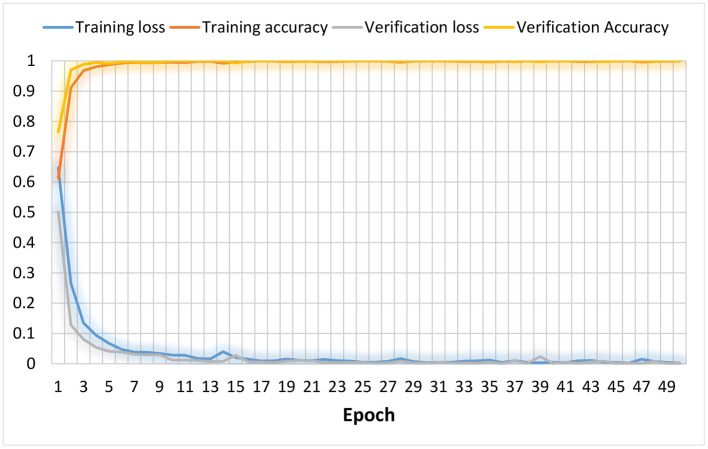
Model optimization curve.

The model used the Adam optimizer, cross-entropy loss function, and 30% of the training set as the validation set. The results of 50 iterations of training are shown in Figure 7. It can be seen from the figure that the training loss dropped rapidly within 5 rounds, and nearly reached a minimum after 18 rounds. The validation loss had the same downward trend as the training loss, and it also quickly approached a minimum, indicating that the model was optimizing quickly. The training and verification accuracy rates rose to 100% in the tenth round, which shows that the model had a strong learning ability. The model had a high recognition rate when the epoch was very small, indicating that it can learn the discriminative characteristics of depression very well. This performance may have been related to the small size of the database.

### 3.5. Comparison With Existing Methods

Due to differences in methodology, datasets, and data usage strategies, it is difficult to fully assess the advantages and disadvantages of various methods based entirely on classification accuracy. However, by comparing indicators such as accuracy, the advantages and disadvantages of various methods can at least be partially evaluated. Table 3 compares the existing state-of-the-art methods with our method in terms of the number of subjects, type and number of channels, research method, number of features, and classification accuracy. Compared to other methods, our method has great advantages. Compared to the other methods, ours has several advantages. First of all, it should be recognized that feature-level fusion (26), multi-variate pattern analysis (27), Case-Based Reasoning Model (29), KNN (31) and our method all have class imbalances (i.e., the difference in the number of subjects between MDD and HC is greater than 1), but nevertheless our method has the highest accuracy. Class imbalances cause models to learn well for a large number of categories but poorly for a small number of categories. Secondly, since depression is classified according to the subject, LOSOCV is more suitable than 10-fold cross-validation, and can ensure that data from the same subject are clustered together. Ten-fold cross-validation may cause data from the same subject to be part of both the training and test sets, which will mislead the classifier to identify the subject itself rather than depression. Therefore, based on this analysis of data balance and test protocol, Brain function networks (33) has the best recognition performance, but our method is only 1.75% less accurate than multi-modal fusion (28). Furthermore, our method need not extract handcrafted features or ensure that the samples are balanced.

**Table 3:** Comprehensive comparison of existing state-of-the-art methods with proposed method.

**Method**	**Subject**	**Channel**	**Feature**	**Protocol**	**Accuracy**
	**(MDD,HC)**				**(%)**
Feature-level fusion (26)	(86, 92)	EEG (3)	60 linear and	Ten-fold CV	86.98
			36 nonlinear features		
Multivariate pattern analysis (27)	(27, 28)	EEG (128)	249 EEG features	LOSOCV	92.73
Multimodal fusion (28)	(81,89)	EEG (3)	6 EEG features	Nested CV	86.64
		and voice(1)	and 15 voice features		
Case-Based Reasoning Model (29)	(86, 92)	EEG (3)	113 EEG features	Ten-fold CV	91.25
SVM (30)	(20, 19)	EEG (64)	3 potential biomarker	Ten-fold CV	89.7
KNN (31)	(92, 121)	EEG (3)	270 features	Ten-fold CV	79.27
Independent component analysis (32)	(13, 13)	EEG (64)	-	-	-
Brain Function Networks (33)	(24, 24)	EEG (64)	LC-CC in theta band	Ten-fold CV	93.31
Correlated Feature Selection (23)	(17, 17)	EEG (128)	10 EEG features	LOSOCV	88.94
Ours	(24, 29)	EEG (128)	-	LOSOCV	90.98

*CV, Cross-Validation; LOSOC, Leave-One-Subject-Out Cross-Validation;MDD, Major Depression Disorder; HC, Healthy Control*.

## 4. Conclusion

This article proposes an MDD deep learning diagnostic framework for depression recognition. Based on the framework, the EEG signals evoked by happy-neutral face pairs were the most discriminative for accurate classification. The method performs well in automatically diagnosing MDD based on EEG signals. The proposed framework makes it possible to directly feed EEG signals into EEGNet for training to improve recognition of MDD in patients. In addition, the method may be of value to the medical device industry for developing diagnostic systems for MDD. Future research will focus on EEG classification of different degrees of depression, and development of a plug-and-play deep learning network to automatic classify the severity of depression.

## Data Availability Statement

The original contributions presented in the study are included in the article/supplementary material, further inquiries can be directed to the corresponding author/s.

## Ethics Statement

Written informed consent was obtained from the individual(s) for the publication of any potentially identifiable images or data included in this article.

## Author Contributions

BL and HC: conceptualization. HC: methodology and resources and data curation. XW: formal analysis and investigation. BL: writing—original draft preparation. KP and XW: writing—review and editing and funding acquisition. All authors have read and agreed to the published version of the manuscript.

## Funding

This work was supported by China Postdoctoral Science Foundation (Nos. 2021M702177 and 2021M690782).

## Conflict of Interest

The authors declare that the research was conducted in the absence of any commercial or financial relationships that could be construed as a potential conflict of interest.

## Publisher's Note

All claims expressed in this article are solely those of the authors and do not necessarily represent those of their affiliated organizations, or those of the publisher, the editors and the reviewers. Any product that may be evaluated in this article, or claim that may be made by its manufacturer, is not guaranteed or endorsed by the publisher.
